# Leaching of Copper Concentrate with Iodized Salts in a Saline Acid Medium: Part 1—Effect of Concentrations

**DOI:** 10.3390/ma16062312

**Published:** 2023-03-13

**Authors:** César I. Castellón, María E. Taboada

**Affiliations:** Departamento de Ingeniería Química y Procesos de Minerales, Facultad de Ingeniería, Universidad de Antofagasta, Av. Angamos 601, Antofagasta 1240000, Chile

**Keywords:** chalcopyrite, leaching, iodide, iodate, seawater, mechanism

## Abstract

One of the main problems in processing chalcopyrite ore with hydrometallurgical methods is its refractoriness, which is due to the formation of a layer that inhibits the contact of the ore with the leaching solution, thus reducing the dissolution rate. The main objective of this paper is to evaluate the leaching potential of iodide ions in copper extraction from chalcopyrite concentrate in an acidic seawater medium. Leaching tests were carried out in glass reactors stirred at 45 °C. Parameters such as iodide salt concentration and acidity were evaluated in ranges of 0–5000 ppm and 0–1.0 M, respectively. According to the results obtained, adding iodide ions to a medium acid enhances the leaching kinetics in the chalcopyrite concentrate, observing that it improves copper extraction at low concentrations of 100 ppm KI compared to high concentrations of 5000 ppm KI. As a result, part of the iodide required to oxidize copper tends to sublimate or is associated with other ions producing iodinated compounds such as CuI. Copper extraction reached 45% within the first 96 h, while at 216 h, it reached an extraction of close to 70% copper. The recovery rate improves at potentials between 600 and 650 mV, while at lower potentials, the copper extraction decreases. The mineral surface was analyzed using SEM/EDS and XRD analyses for the identification of precipitates on the surface, finding porous elemental sulfur and precipitated jarosite. An increase in iodide ions improves the leaching kinetics in the chalcopyrite concentrate, observing that it improves copper extraction at low concentrations of 100 ppm KI compared to high concentrations of 5000 ppm KI. As a result, part of the iodide required to oxidize copper tends to sublimate or is associated with other ions producing iodinated compounds such as CuI. Copper extraction reached 45% within the first 96 h, while at 216 h, it reached an extraction of close to 70% copper. The recovery rate improves at potentials between 600 and 650 mV, while at lower potentials, the copper extraction decreases. The mineral surface was analyzed using SEM/EDS and XRD analyses for the identification of precipitates on the surface, finding porous elemental sulfur and precipitated jarosite.

## 1. Introduction

Today, around 20% of copper-bearing ores are treated with the hydrometallurgical process, which stands out as a process with low pollutant emissions and lower energy consumption [1,2]. Copper hydrometallurgy addition to achieving good results in the treatment of low-grade ores [3,4,5] has now been studied as an alternative to the treatment of complex copper sulfide concentrates [6,7]. Despite the development of processes to obtain copper from the primary sulfide ore, its application on an industrial scale has been limited [3,8]. Even after almost 50 years, a passivation layer is still the main reason for its scarce application [8,9].

Chalcopyrite (CuFeS_2_) is the primary copper sulfide mineral that represents approximately 70% of the world’s known copper reserve [10,11,12,13] and is present in secondary resources [14]. It also coexists with pyrite (FeS_2_) and other secondary or supergene minerals such as covellite (CuS), chalcocite (Cu_2_S), or digenite (Cu_2_S) and even with minerals such as bornite (Cu_5_FeS_4_) and enargite (Cu_3_AsS_4_).

Although the term passivation had been used since the 1830s [15,16], it was not until the late 1970s that the slow leaching kinetics of chalcopyrite was related to the formation of a layer [17]. However, despite the wide variety of studies, there is still no clear conclusion on the process of formation and composition of this film. Among the hypotheses found are the formation of elemental sulfur (S_0_) [18,19,20,21], disulfides (S_2_^2−^) [20,22,23], polysulfides (S_n_^2−^) [20,24], metal-deficient sulfide [25,26], copper-rich polysulfides [27], and formation of iron hydroxides or jarosites on the surface of the ore [28,29].

Several methods have been used to increase reaction rates and break or avoid this passivation layer in leaching systems, including (i) leaching with powerful oxidizing agents such as O_3_ [30,31,32], H_2_O_2_ [6,33,34,35], potassium permanganate, and potassium dichromate [36,37,38], (ii) leaching with high pressure and temperature [34,39,40,41], and (iii) bacterial leaching [11,14,42,43], but these improvements are accompanied by limitations, mainly due to the high cost of oxidizers, the corrosive environments they provide and even low copper recoveries [39,44]. These kinds of considerations result in the hydrometallurgical route not being widely used in the dissolution of copper from primary sulfide ore, mainly because of slow leaching kinetics caused by the crystal structure of chalcopyrite (face-centered tetragonal lattice), which makes its lattice energy too high to break S-Cu-Fe bonds [23,45].

The use of halide oxidizers such as iodide in a leach presents an alternative in copper recovery. It is currently widely used in gold and silver leaching [46,47,48]. The use of this type of iodide salt dissolves the sulfide minerals due to the high proton activity created with the mixture between acid and oxygen [49]. The use of iodide ions (I^−^) can be particularly attractive because they improve the recovery of copper ores [50] and offer an alternative to the ferric ion leaching process [51]. Even a mixture of these two lixiviants results in higher dissolution created by the duplex (Fe^3+^/I^−^) [35,50,52,53,54]. Konyratbekova, Baikonurova [49] indicates that the use of leaching agents such as Cl_2_, Br_2_, and I_2_ salts, together with iodide ions from the KI salt, significantly increases the probability of dissolution of metal sulfides, in particular iron sulfides such as pyrite (FeS_2_).

**Figure 1:**
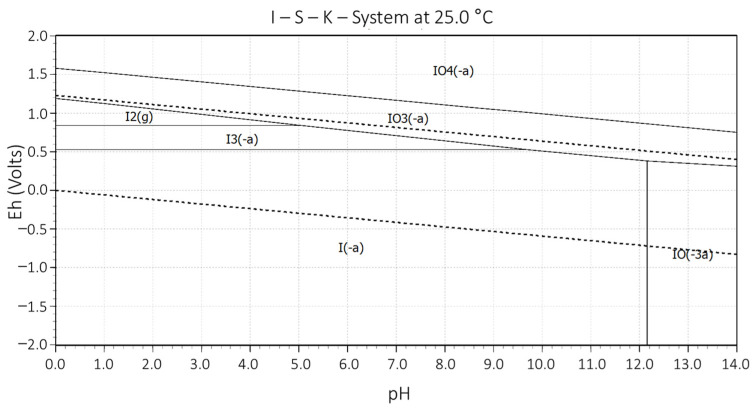
Eh–pH diagram system I-O. [I] = 0.001 M.

Although elemental iodine is very insoluble in water, the formation of a copper complex with iodide and chloride ions can increase the solubility because they can act as Lewis bases. If the concentration of the iodide ion is increased in an iodide–iodide mixture (I_2_/I^−^) with a pH less than 9, then the solubility of the overall iodine increases through the formation of the triiodide ion, I_3_^−^ [48]. This triiodide ion is the result of the reaction of iodine with the produced iodide ion (Reaction (1)) or by hydrolytic reactions of iodine [55] in which the triiodide ion is the fundamental leaching agent that functions as a catalyst for the principal leaching reaction of the system (Reactions (1)–(3)). In Figure 1, the stability region of the triiodide ion is shown with approximate values between 0.5 and 1.3 V, a range in which it is the most stable of the polyiodides formed.

(1) $$I_{2\left(s\right)}\to I_{2\left(aq\right)}$$

(2) $$I_{2\left(aq\right)}+I^{-}\to I_{3}^{-}\;\Delta G{{}^{\circ}}_{318}(\mathrm{kJ})=-15.877$$

(3) $$I_{3}^{-}+2e^{-}\to 3I^{-}\;\Delta G{{}^{\circ}}_{318}(\mathrm{kJ})=-102.852$$

In addition, the triiodide ion can favor the oxidation of ferrous ores (Fe^2+^) converting them to ferric iron (Fe^3+^), where the latter will oxidize the iodide that will subsequently allow the leaching of chalcopyrite through the formation of triiodide ions [56].

Ideally, the salts used as leaching agents should be low-cost or recyclable, selective, and compatible with the subsequent recovery process. Specifically, the caliche ore from northern Chile, a mineral with high concentrations of nitrate and iodine, contains the largest deposits of iodide in the world [57]. These deposits have shown that in addition to being the primary source of natural nitrate and having many iodine salts, they also contain salts such as bromides and chlorides [58] that offer a high oxidation power towards sulfides when added to an acid medium.

Therefore, this paper carefully discussed and studied the leaching process of chalcopyrite concentrate with different concentrations of potassium iodide salts as a leaching agent and sulfuric acid using seawater and fresh water. All of the above were carried out at atmospheric pressure and moderate temperature (≤45 °C).

## 2. Materials and Methods

### 2.1. Mineral Sample and Reagents

Chalcopyrite concentrate for the tests was obtained from a Chilean mining company. The sample is from a porphyritic deposit with a potassic alteration type, where the mineralization of the primary zone (chalcopyrite) is present as disseminations with grain size from microns to millimeters and with 0.8% Cu_T_. The particle size used in this study was P_80_ of 70.66 µm. The particle size distribution was determined using Microtrac S3500. The chemical composition was determined using inductively coupled plasma atomic emission spectroscopy (ICP-AES), and the mineralogical content was determined with a quantitative evaluation of the minerals using scanning electron microscopy (QEMSCAN) model Zeiss EVO 50 (Zeiss, Oberkochen, Germany).

The samples were characterized with scanning electron microscopy (SEM) using the Zeiss EVO MA10 model equipment, which has an Oxford model X-maxN 20 SDD energy dispersive X-ray analyzer (EDS), and with the X-ray diffraction (XRD) equipment Shimadzu XRD 6100 (Shimadzu Corporation, Kyoto, Japan). Chord length measurements were determined to determine the particle size using the Mettler Toledo Particle Track G400 focused beam reflectance measurement (FBRM) technique.

Potassium iodide (99.0% absolute, Merck, Darmstadt, Germany), potassium iodate (99.0% absolute, Merck, Darmstadt, Germany), sulfuric acid (95–97%, Merck, Darmstadt, Germany), and sodium chloride (99.9%, Merck, Darmstadt, Germany) were used in the leaching tests at analytical grade. Seawater was obtained 200 m from the coast in San Jorge Bay, Antofagasta, Chile. The seawater (pH = 7.1) was passed through a quartz sand filter (50 µm) and a mechanical polyethylene filter (1 µm) to remove insoluble particulate matter.

### 2.2. Procedure of the Leaching Experiments

**Figure 2:**
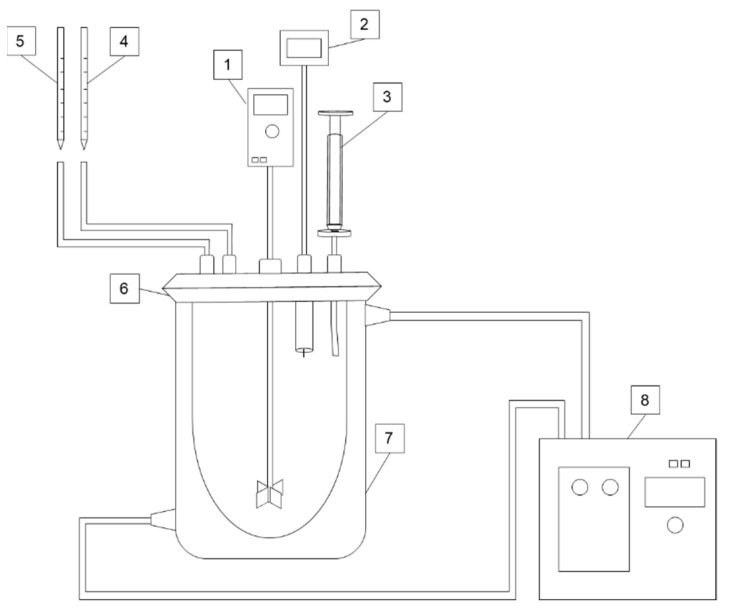
The schematic diagram of the leaching setup: 1. overhead stirrer; 2. pH and ORP probe; 3. sampling tube, equipped with syringe and syringe filter; 4. H_2_SO_4_; 5. oxidant; 6. sealed lid; 7. Jacketed reactor; and 8. water bath.

Concentrate leaching tests were performed in 2 L jacketed glass reactors (See Figure 2). Each reactor was loaded with 1 L of leaching solution (sulfuric acid, seawater, iodide potassium or iodate potassium, and sodium chloride) and was sealed with a film to avoid evaporation. Once the solution reached the desired temperature, 50 g of the solid sample was added to the reactor. Before the leaching tests, the concentrate sample was washed with distilled water and acetone (C_3_H_6_O) with the purpose to remove any flotation reagents left used in the process of concentration. The pulp was stirred to obtain a homogenous mix using a propeller with a rotation speed of 450 rpm. A 10 mL aliquot of the solution was taken periodically during the test for copper analysis using the Atomic Absorption Spectroscopy method (AAS). Redox potential (ORP) and pH were measured throughout the test with a portable Hanna meter (model HI991003). All experiments were performed in triplicate. The solid residues were carefully filtered, washed with distilled water, and dried at 60 °C to constant weight. A sample for mineralogical characterization and particle size determination was taken.

## 3. Results and Discussions

### 3.1. Mineral

**Figure 3:**
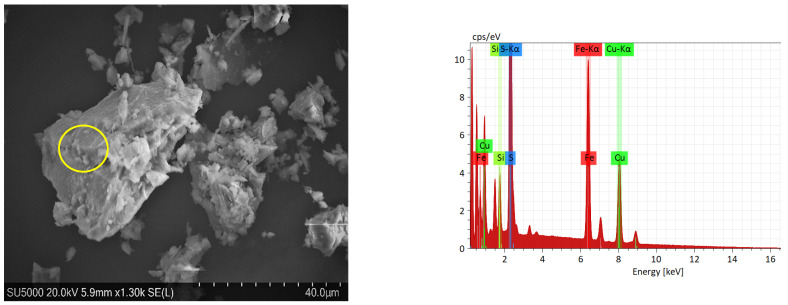
SEM and EDS micrograph of the chalcopyrite concentrate. Yellow circle shows EDS punctual analysis.

**Figure 4:**
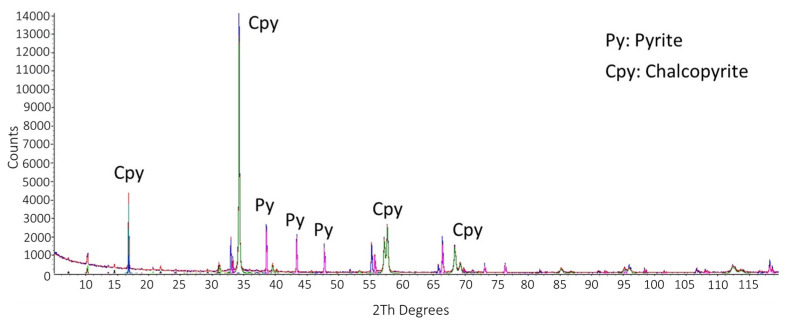
X-ray diffraction pattern of the chalcopyrite concentrate.

Table 1 shows the chemical analysis and mineralogical information were mainly composed of chalcopyrite (61.51 wt. %) and pyrite (23.3 wt. %), with small amounts of covellite, chalcanthite, sphalerite, and molybdenite. The gangue minerals were quartz, dolomite, muscovite, and albite. Based on these data, the sample is composed of 63% copper sulfides followed by 24.3% of other sulfides. In addition, Figure 3 shows an SEM micrograph of the concentrate with its respective EDS and DRX in Figure 4.

A typical analysis of the seawater is presented in Table 2, which was obtained using different analytical techniques (ICP-AES, atomic absorption spectrometry-AAS, Volumetric, and Gravimetric Analysis).

### 3.2. Effect on Iodide Ions Concentration

**Figure 5:**
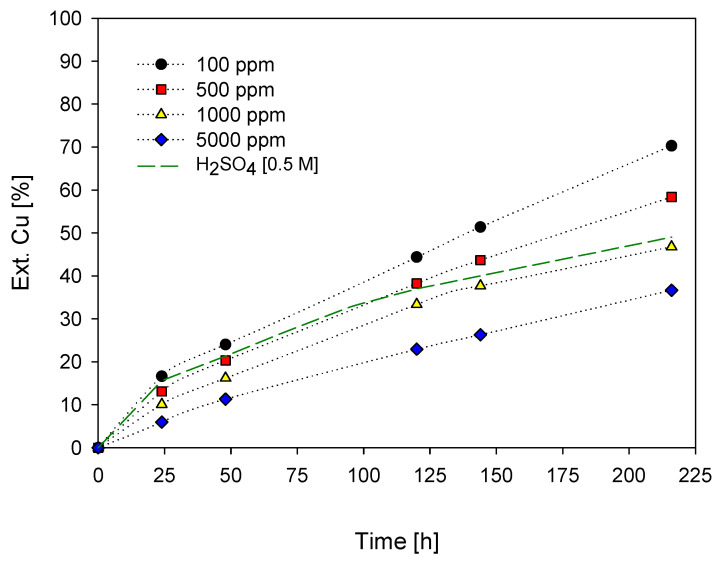
% Cu Ext. vs leaching time. [KI] = (●) = 100 ppm; (■) = 500 ppm; (▲) = 1000 ppm; (◆) = 5000 ppm. [Conditions: H_2_SO_4_ = 0.5 M, T = 45 °C, 50 g of sample in 1000 mL of seawater solution, 450 rpm].

Figure 5 shows the copper concentrate leaching results for 216 h at 45 °C using the addition of potassium iodide in the range of 100 to 5000 ppm with a concentration of 0.5 M H_2_SO_4_. It should be mentioned that leaching systems with lower iodide concentrations (<50 ppm) were not included as these gave lower copper extractions (<30%), and their effect on the ore could be confused with that of sulfuric acid.

It is observed, according to the graph, that the highest extraction was obtained with 100 ppm of potassium iodide. The copper recovery increased from 36.7 % to 70.3 % after 200 h of leaching when the KI concentration decreased from 5000 ppm to 100 ppm. In addition, the extraction curves do not show that the steady state has been achieved for these systems, which could indicate no formation of any elemental sulfur passivation layer or precipitates on the surface of the particles, and then that higher leaching times would allow for obtaining better extractions. Likewise, the formation of iron hydroxide is discarded because of pH values lower than 2 [60]. After this pH, the transfer of electrons through the formed layer of iron hydroxides is inhibited.

The low copper extraction at a high KI concentration (5000 ppm) is shown in reaction 4, where the iodide ions are oxidized to elemental iodine by sulfuric acid. In turn, the low solubility of the iodide ions allows them to be released from the solution in gaseous form and condense on the reactor walls. The oxidation of the iodide ions is corroborated by the rapid coloration of the reactor in violet-red. Thus, at higher concentrations of H^+^ and I^−^, the oxidation of I^−^ by the oxygen present, generated with the agitation, occurs to a greater extent.

(4) $$5H_{2}SO_{4}+8KI\to 4H_{2}O+H_{2}S+4I_{2\left(g\right)}+4K_{2}SO_{4}\;\Delta G{{}^{\circ}}_{318{}^{\circ}C}(\mathrm{kJ})=-164.557\;∆H{{}^{\circ}}_{318}(\mathrm{kJ})=23.351$$

In turn, the presence of iodide ions in the system also allows triiodide ions to form in the solution, although to a lesser extent (Reactions (5) and (6)). This formation occurs with a decrease in pH.

(5) $$I_{2\left(aq\right)}+I^{-}↔I_{3}^{-}\;\Delta G{{}^{\circ}}_{318{}^{\circ}C}(\mathrm{kJ})=-15.877\;∆H{{}^{\circ}}_{318}(\mathrm{kJ})=-12.755$$

(6) $$6I^{-}+O_{2\left(g\right)}+4H^{+}\to 2I_{3}^{-}+2H_{2}O\;\Delta G{{}^{\circ}}_{318{}^{\circ}C}(\mathrm{kJ})=-262.087\;∆H{{}^{\circ}}_{318}(\mathrm{kJ})=-308.675$$

**Figure 6:**
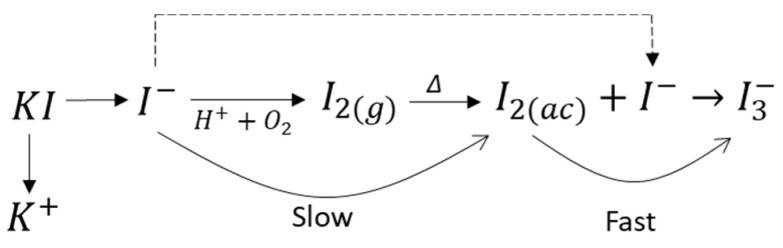
Schematization of the formation of the triiodide ion.

Oxidation and formation of triiodide ions are observed in the schematic diagram in Figure 6, where potassium iodide salts dissociate into potassium cations and iodide ions upon contact with water. Iodide ions can release themselves and come out of the solution in the presence of some heat. However, the leaching system is closed, that is, the gas/air leakage is minimal, so the gaseous iodine that sublimates and crystallizes on the reactor walls as elemental iodine, with the action of Henry’s law, returns to solution as aqueous iodine. Once in solution, the aqueous iodine reacts rapidly with the iodide ion from the dissociation of KI to form the new oxidant triiodide ion. The formation of elemental iodine can be defined as a pseudo-order reaction, especially because the reactants (acid and iodide) are in high excess; however, the generation of the triiodide ion is rapid upon reaching equilibrium.

However, the reaction kinetics in reaction 6 are complicated and slow due to the participation of 11 particles where oxygen, with 4 electrons, acts in stages consuming hydrogen ions and forming intermediate species such as O^2−^, HO_2_^•^, H_2_O_2_, and OH^•^. These intermediates usually limit the reaction rate. In addition, a low copper recovery at higher iodide concentrations could have resulted from the formation of more inactive species that can form under conditions of higher iodide concentrations.

**Figure 7:**
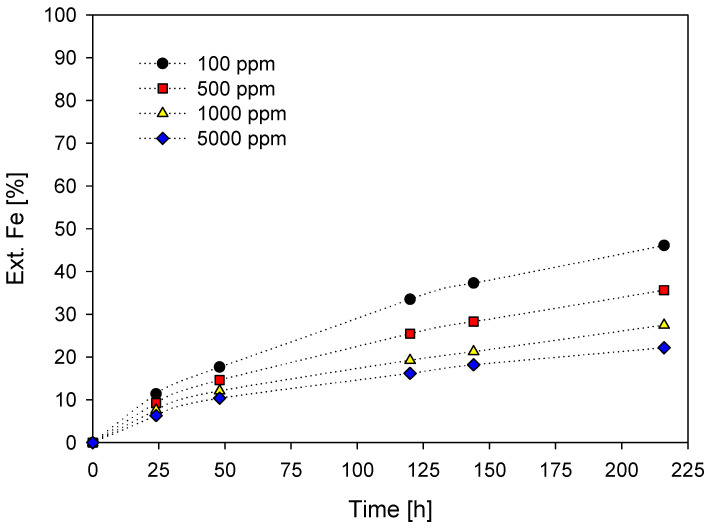
% Fe Ext. vs leaching time. [KI] = (●) = 100 ppm; (■) = 500 ppm; (▲) = 1000 ppm; (◆) = 5000 ppm. [Conditions: H_2_SO_4_ = 0.5 M, T= 45 °C, 50 g of sample in 1000 mL of solution of seawater, 450 rpm].

Likewise, the extraction profile of iron after the addition of potassium iodide is shown in Figure 7, noting that iron extraction follows a similar trend to copper, with a higher resolution of 46.1% Fe observed at 100 ppm iodide for 216 h, in contrast to the simultaneous addition of 5000 ppm iodine compound to 22.2%. According to the results, the copper extraction is higher than the iron extraction with the action of the pyrite–chalcopyrite galvanic pair, also shown in other works [61,62,63,64,65,66]. In addition to the dissolution of iron from the chalcopyrite ore, other minerals that increase the extracted value of iron are the gangue minerals, where iron is present; however, according to the mineralogy of the concentrate, these percentages are minimal.

**Figure 8:**
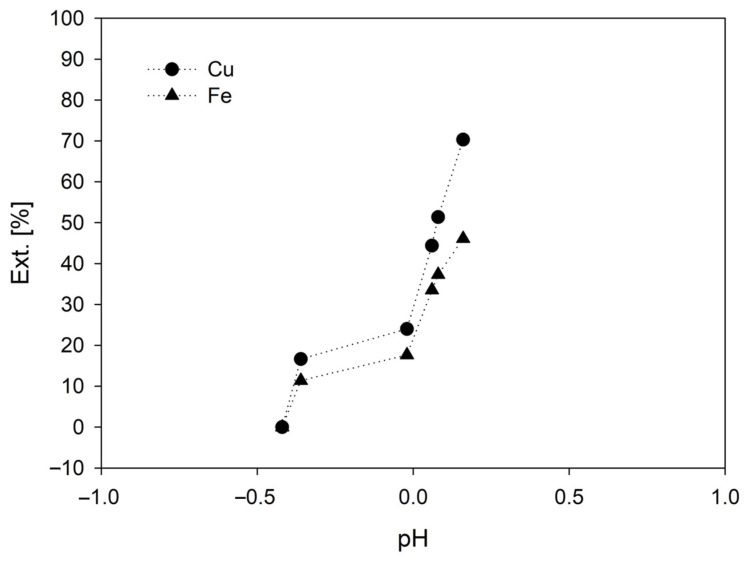
Effect of pH on metal extraction [Conditions: H_2_SO_4_= 0.5 M, 45 °C, 50 g of sample in 1000 mL of seawater solution, 450 rpm].

pH is one of the main parameters for copper leaching that is generally performed under strongly acidic conditions (pH < 2) to limit the precipitation of extracted copper ions (Cu^2+^) and Fe^3+^. Figure 8 shows that when the pH ranged from 0 to 0.5, the extraction of both metals increased due to the better leaching conditions in the system (100 ppm of KI and 0.5 M of H_2_SO_4_). Again, the graph shows that in both metals, their dissolution does not stop, as confirmed by the extraction curves, where positive slopes are maintained throughout both metals (see Figure 5 and Figure 7). Likewise, it is observed that the iron extraction curve, after having a pH greater than 0, begins to decrease its extraction, separating itself from the copper extraction curve. Thus, this suggests that the effectiveness of its extraction decreases as the pH increases. The above is due to a decrease in the acidity of the solution.

The slight increase in pH during leaching is due to the dolomite present as well as biotite, which, according to studies, is one of the most acid-reactive silicates [67,68], while to a lesser extent muscovite and albite [69]. Although the latter is less soluble in acid, grain size plays an important role in reactivity. The dissolution of gangue during the acid interaction will allow the release of elements such as Ca, Mg, Na, K, and Si, which will interact with the minerals to form new products that will precipitate on the surface of the particles. It should be noted that the high concentration of acid used in this test is because it has shown favorable results in the extraction of copper from this concentrate in previous works [70,71,72].

**Figure 9:**
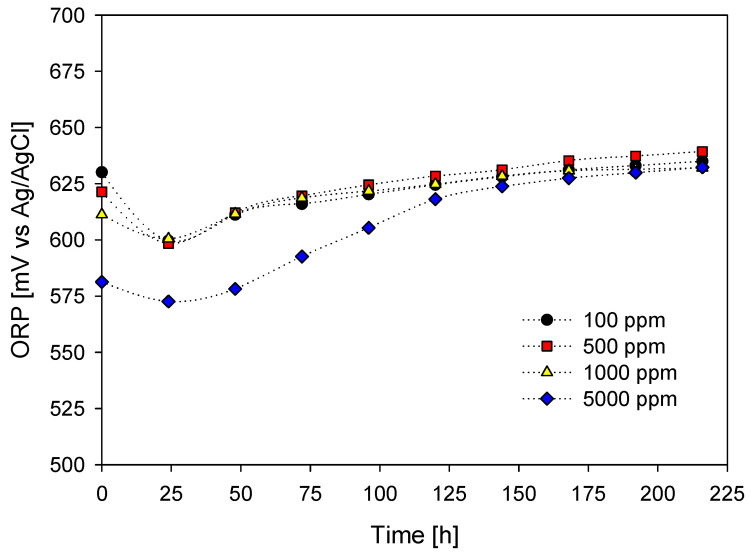
ORP effect vs time on metal extraction. [KI] = (●) = 100 ppm; (■) = 500 ppm; (▲) = 1000 ppm; (◆) = 5000 ppm. [Conditions: H_2_SO_4_ = 0.5 M, 45 °C, 50 g of sample in 1000 mL of seawater solution, 450 rpm].

In the case of ORP behavior, shown in Figure 9, all the curves have a slightly ascending and almost linear slope. The above is because in an acidic environment, oxygen in the air can release iodine from iodide-containing solutions, and the generated iodine dissolves easily in aqueous iodide solutions (Reactions (7) and (8)).

(7) $$I_{\left(ac\right)}^{-}+\frac{1}{2}O_{2\left(g\right)}+2H_{\left(ac\right)}^{+}\to I_{2\left(s\right)}+H_{2}O_{\left(l\right)}$$

(8) $$I_{2\left(s\right)}+I_{\left(ac\right)}^{-}\to I_{3\left(ac\right)}^{-}$$

In all curves, there is an initial decrease and then a continuous increase during the leaching time. Studies show that the leaching rate of chalcopyrite in acidic solutions is very dependent on the redox potential of the system [22,73,74].

The curve with 5000 ppm iodide had lower redox potential and, after some time, was closer to the range of the others. The low initial redox potential value was mainly the result of the high amount of iodide salt added at the beginning of the test that, together with the seawater, allows the sulfuric acid to act as a reducer and not as an oxidizer.

After the leaching time, in all the tests, the redox potential of the curves achieved values close to 630 mV (Ag/AgCl), which proves that they are between the ion zones (I_3_^−^ and I^−^) according to Figure 1. Likewise, several authors have identified a window between 610 to 640 mV (Ag/AgCl), where they observed the highest recovery of copper from chalcopyrite ore. Outside this potential window, chalcopyrite is in its bistable or passive state [61,75,76,77,78,79,80].

**Figure 10:**
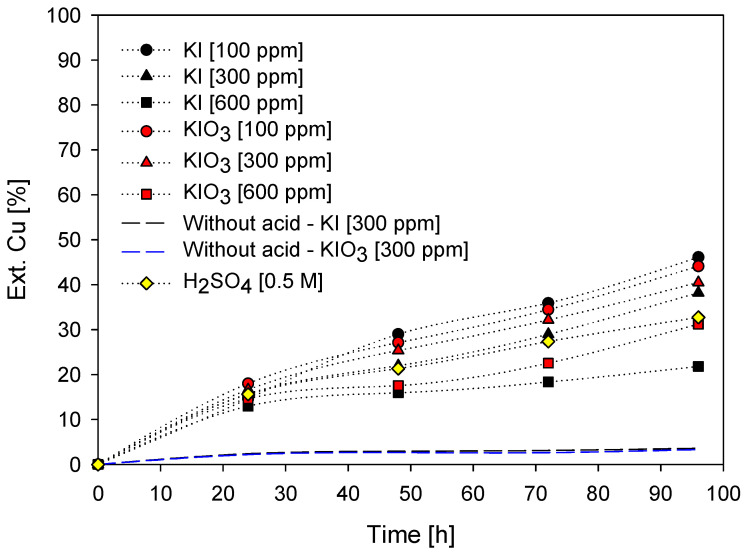
% Cu. Ext. vs time of leaching [Conditions: H_2_SO_4_ = 0.5 M (unless otherwise indicated), 45 °C, 50 g of sample in 1000 mL of seawater solution, 450 rpm].

According to the copper extraction obtained in Figure 5, where at low iodide concentrations more favorable copper extractions are obtained, leaching systems with concentrations of 100, 300, and 600 ppm KI with 0.5 M H_2_SO_4_ at a temperature of 45 °C were performed. These curves can be seen in the graph in Figure 10. Likewise, it included the addition of the KIO_3_ salt to determine and compare the effect of both salts on copper extraction.

According to the figure, 100 ppm of KI presents an extraction of 45.2% of Cu in 96 h. Likewise, the KIO_3_ salt with the same concentration obtains an extraction of 44.1 % of Cu. The above concludes that at a concentration of 100 ppm of salt, potassium iodide (KI) has greater importance in copper extraction than potassium iodate salt (KIO_3_). This slight increase in extraction is due to the higher mass percentage of iodine in the KI compound (76.4%) as opposed to KIO_3_ (59.3%). In contrast, at a concentration of 300 ppm, KIO_3_ shows a higher copper extraction, unlike the KI salt. However, for both salts, there is an unfavorable effect on copper extraction when concentrations above 100 ppm are used.

Likewise, it is observed that, at the end of the leaching time, KIO_3_ shows better extraction at 300 ppm, and extraction increases even more at a concentration of 600 ppm, in contrast to the use of KI. This improvement in copper extraction with KIO_3_ salt at concentrations higher than 100 ppm could be due to several factors, among which are the following:

1. As a result of the oxygen present in the salt. Potassium iodate, when heated, decomposes into KI and O_2_, according to Reaction (9) [81]. Oxygen may also oxidize the mineral.

(9) $$KIO_{3}\overset{\Delta }{\to }KI+O_{2}$$

2. The iodate ion (IO_3_^−^) acts as a better oxidizing agent in an acidic medium because it has a more positive electrode potential (Reaction (10)).

(10) $$IO_{3}^{-}+6H^{+}+6e^{-}\to I^{-}+3H_{2}O\;\Delta G{{}^{\circ}}_{45{}^{\circ}C}(\mathrm{kJ})=-633.059\;E{}^{\circ}=1.1\;V$$

3. Furthermore, for the reaction between the ferrous ion (Fe^2+^) found in the acid solution and the iodate ion forms a ferric ion (Fe^3+^), which is a new oxidant in the system (Reaction (11)). Fe sites are preferentially oxidized to Cu sites, leading to the formation of iron oxides and ferrous hydroxides on the chalcopyrite surface [82]. However, this reaction is less favorable.

(11) $$IO_{3}^{-}+6H^{+}+5Fe^{2+}\to 0.5I_{2}+3H_{2}O+5Fe^{3+}\;\Delta G{{}^{\circ}}_{45{}^{\circ}C}(\mathrm{kJ})=-199.154$$

4. Finally, another important feature reported in studies is that iodate (I^5+^O_3_^−^) is known to be retained on solids such as phyllosilicates, metal surface oxyhydroxides, etc., even when found in acidic environments [83] so that the salt reaction is incomplete.

It is important to comment that the tests in the acid-free condition resulted in extractions of less than 5% Cu. The latter highlights the importance of the presence of sulfuric acid with the iodized salt since in an acid-free environment, iodine acts as a reducing agent and not as an oxidizing agent. Therefore, the presence of the acid together with the salt allows for obtaining copper extractions from a concentrate.

Figure 5 and Figure 10 show that iodized salts in excess or at high concentrations can oxidize to elemental iodine and sublimate. When found in low concentrations, in addition to being a good oxidizer, they can also precipitate on the mineral grains in a compound known as cuprous iodide (CuI). The iodide or iodate ion, present in the solution along with Cu^2+^, reacts to form cupric iodide (CuI_2_), a soluble ion. This compound rapidly decomposes into iodine and cuprous iodide, making the latter insoluble (see Reaction (12)). Here the characteristic color of iodine occurs due to the release of I_2_ gas.

(12) $$2CuI_{2}\to 2CuI+I_{2\left(g\right)}\;\Delta G{{}^{\circ}}_{318{}^{\circ}C}(\mathrm{kJ})=-87.725\;\Delta H{{}^{\circ}}_{318}(\mathrm{kJ})=-41.161$$

In addition to the above reaction, CuI formation can occur by subsequent chemical reactions with iodide ions (Reactions (13)–(15)).

(13) $$Cu^{2+}+I^{-}+e^{-}\to CuI\;\Delta G{{}^{\circ}}_{318{}^{\circ}C}(\mathrm{kJ})=-82.98\;E_{0}\;(V)=0.861$$

(14) $$3Cu^{2+}+I_{3}^{-}+5e^{-}\to 3CuI\;\Delta G{{}^{\circ}}_{318{}^{\circ}C}(\mathrm{kJ})=-351.79\;E_{0}\;(V)=0.730$$

(15) $$Cu^{2+}+I_{2}+3e^{-}\to CuI+I^{-}\;\Delta G{{}^{\circ}}_{318{}^{\circ}C}(\mathrm{kJ})=-201.71\;E_{0}\;(V)=0.697$$

**Figure 11:**
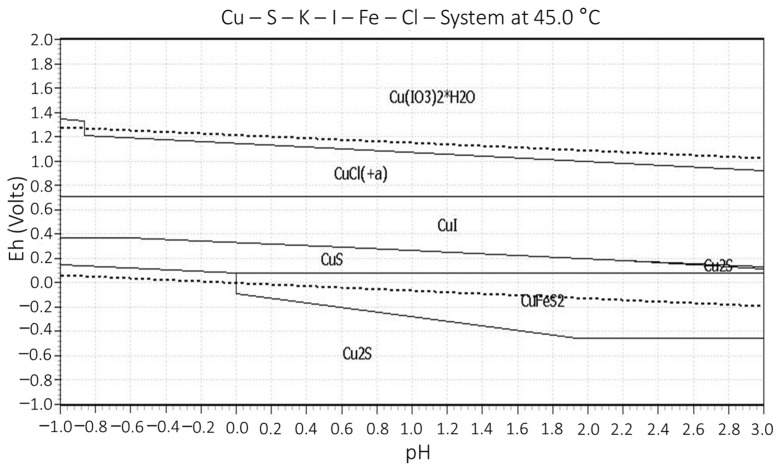
Eh vs pH diagram for K-I-Cu-Fe-S-Cl system at 45 °C.

Figure 11 allows us to check the stability of CuI in the range of 400–700 mV. In this range is the potential of the leaching solutions. However, it is also observed from the graph that above this value, copper is in solution as CuCl^+^/Cu^2+^.

**Figure 12:**
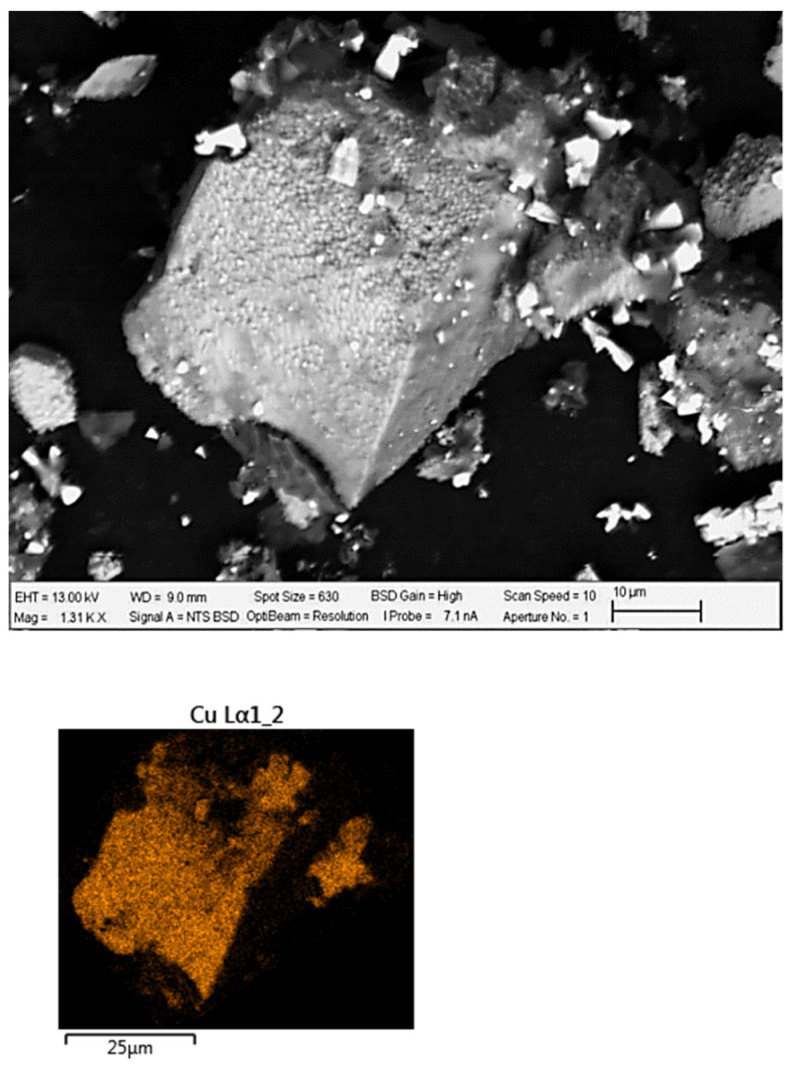

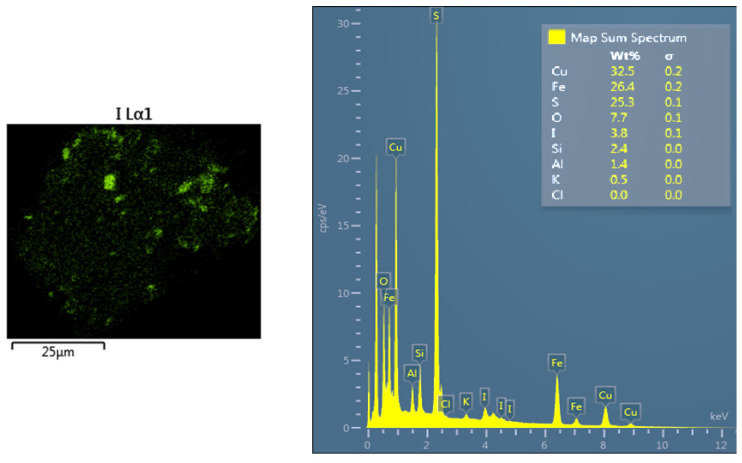
SEM and EDS micrograph of a CuFeS_2_ particle with element distribution (Cu: orange, I: green). Test conditions: KI = 100 ppm, H_2_SO_4_ = 0.5 M, T= 45 °C in seawater.

The presence of some CuI precipitates on the surface of the chalcopyrite particles can be confirmed with SEM and EDS, as shown in Figure 12.

**Figure 13:**
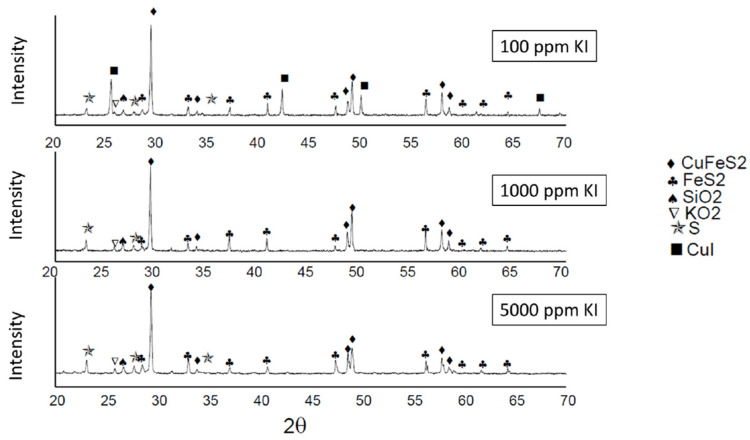
Diffractograms of the leaching tests using 100, 1000, and 5000 ppm KI with 0.5 M H_2_SO_4_.

Chalcopyrite grains observed in microscopy showed some percentage of iodine due to the formation and precipitation of CuI. The formation of CuI complexes and the absence of copper chloride results in better stability of the iodide ions [49,84,85]. This assertion was demonstrated with the EDS analysis of chalcopyrite grains and X-ray diffractograms shown in Figure 13. In addition, at lower pH, the concentration of OH^−^ ions decreases, resulting in more positive sites available for I^−^ anions to occupy [86].

Furthermore, the presence of CuI compounds is detected in the X-ray diffractograms in Figure 13. These ripples belong to the following samples: 100 ppm KI–0.5 M H_2_SO_4_, 1000 ppm KI–0.5 M H_2_SO_4_, and 5000 ppm KI–0.5 M H_2_SO_4_. Therefore, the diffractograms confirm that the presence of this compound occurs when adding 100 ppm of KI, but at 5000 ppm it is not detected, which assumes sublimation as I_2_ gas.

### 3.3. Sulfuric Acid Concentration

In addition to investigating and discussing the use of iodized salt concentrations with 0.5 M H_2_SO_4_ and seawater, the effect of acidity in leaching systems was also studied at a fixed concentration of 100 ppm KI. This concentration of KI salt was chosen because it presented the most favorable copper extraction (45.2%) using seawater and a temperature of 45 °C (see Figure 5 and Figure 10).

**Figure 14:**
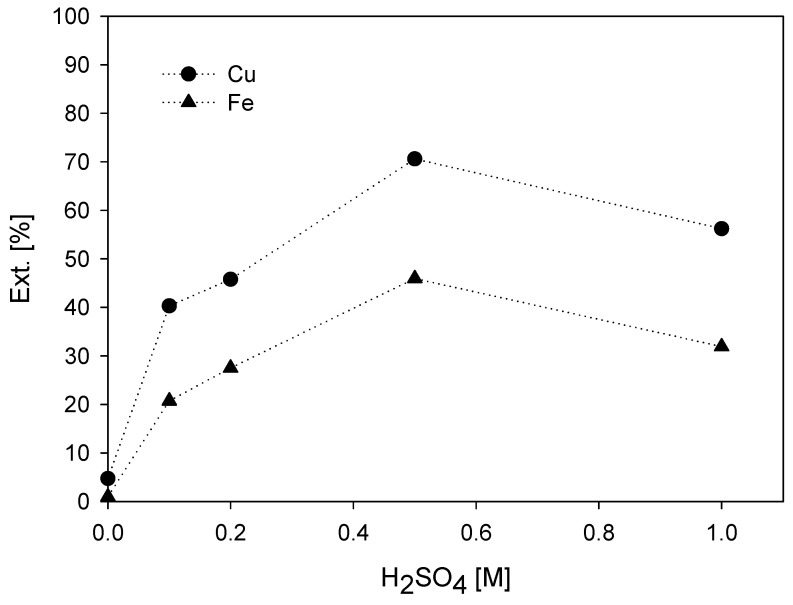
Effect of acid concentration on metal extraction. (●) = Cu; (▲) = Fe. [Test conditions: KI = 100 ppm, T= 45 °C in seawater, 450 rpm].

The graph in Figure 14 shows the results from leaching 50 g of chalcopyrite concentrate with a leaching time of 192 h and a concentration of 100 ppm KI at 0.0 M, 0.1 M, 0.2 M, 0.5 M, and 1.0 M, respectively.

According to the graph, copper extraction increases with increasing acidity. However, the extraction decreases with doses higher than 0.5 M H_2_SO_4_.

The copper extraction at the end of the leaching time was 40.3% and 45.8% using an acid concentration of 0.1 M and 0.2 M, respectively. With 0.5 M sulfuric acid, the extraction was 70.6%. However, with 1.0 M H_2_SO_4_, belonging to the highest acid concentration, there is a lower extraction of 56.2% copper.

In addition to the sulfuric acid systems, the figure shows a leaching system with no acidity and 100 ppm KI. This system shows extractions of up to 4.7% at the end of the leaching time. The low copper extraction in the absence of sulfuric acid suggests that the added iodide reacts and transforms into a reducing agent according to its dissociation and subsequent oxidation. Therefore, the presence of sulfuric acid has a positive effect in providing the right environment for chalcopyrite dissolution. However, according to the graph, a high amount of sulfuric acid is detrimental to extraction. At concentrations higher than 0.5 M, the effect starts to be counterproductive by giving a higher loading capacity to the leaching solution and sulfating the remaining copper resulting in a slower copper dissolution rate. In addition, a high initial concentration of sulfuric acid can function as a neutralizing agent, just as a lack of acid can cause iron precipitation [40]. Other research indicates that a higher sulfate concentration may produce several diffusion layers around minerals, such as pyrite and chalcopyrite, which will decrease leaching rates and activation energies [87,88].

The high copper extraction (70.6%) with 100 ppm KI and 0.5 M H_2_SO_4_ may be due to the free acidity of the solution favoring the formation of the ferric ion (Fe^3+^). The ferric ion provides a two-step synergistic effect where the iodide is oxidized to iodide by the ferric while in a second step, iodine oxidizes the chalcopyrite converting it back to iodide, i.e., the generated elemental iodine reacts with the remaining iodide ions, so that triiodide ions are generated in the solution. The triiodide ions also function as catalysts for the reaction and thus repeat this cycle of redox reactions [52,56]. The formation of the ferric ion, a new oxidant in the system, comes from the amount of pyrite in the initial sample. However, this is inconsistent with the interaction of the galvanic couple.

In the case of iron extraction, it is like that of copper. At concentrations above 0.5 M, iron extraction decreases. A higher dose of sulfuric acid at the optimum or necessary concentration will allow a high net acid consumption due to the release of contaminants or other ions from the gangue. There may even be an increase in impurities by dissolution and formation of new, more complex minerals where they are favorable to the given environment. In addition, the anodic oxidation of pyrite decreases with increasing concentration of sulfate ions from increased H_2_SO_4_. This decrease can be attributed to the presence of sulfate ions leading to the formation of electrochemically less reactive iron sulfate complexes.

**Figure 15:**
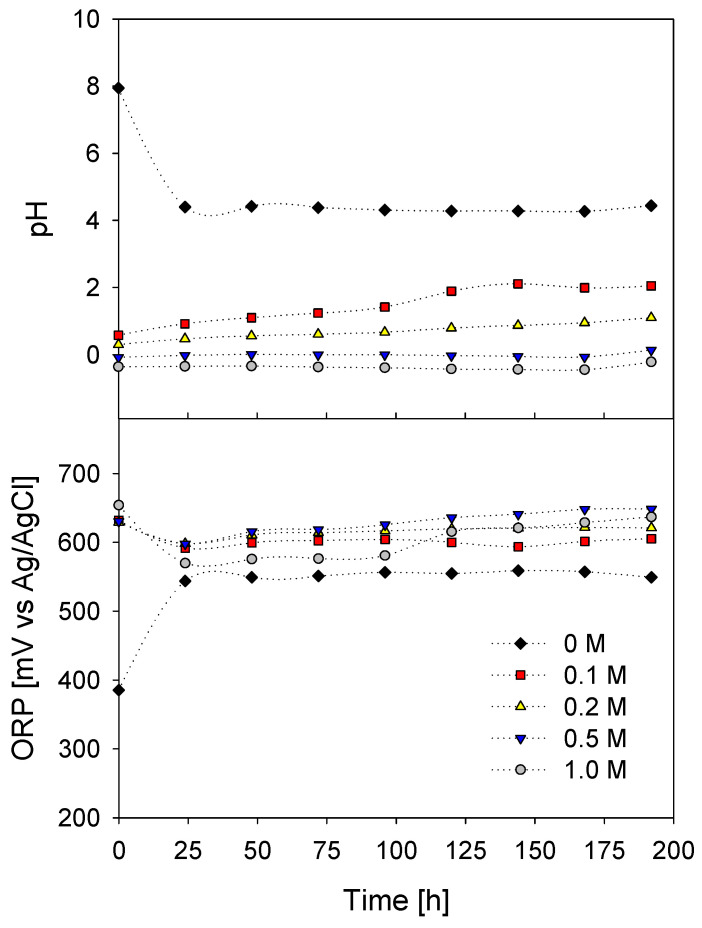
pH–ORP vs Leaching time. [H_2_SO_4_] = (◆)= 0 M; (■) = 0.1 M; (▲) = 0.2 M; (▼) = 0.5 M; (●) = 1.0 M. [Conditions: KI = 100 ppm, T = 45 °C, 50 g of sample in 1000 mL of solution with seawater, 450 rpm].

The pH values close to 0 and 1 in the leaching systems, shown in Figure 15, show that the concentration of protons (H^+^) is high and stable. On the other hand, in the absence of acidity, at the beginning of the test, the pH is maintained in a range of 7.9 to 4.4, and after 25 h of leaching, it stabilizes at pH = 4.3. This initial pH value agrees with that of seawater in a leaching system (7.5–8.5), but its decrease is due to the abrasion between the mixture of sulfides such as chalcopyrite and pyrite and the solution, which reflects pH values between 4 and 5 at that moment that the aqueous equilibrium is established.

Likewise, the increase in the oxidizing potential of the iodide ions concerning acidity contributes greatly to copper leaching (see graph in Figure 15). ORP values found between 500 and 650 mV (Ag/AgCl) promote chalcopyrite leaching and coincide with the stability zone in the triiodide ion as an oxidant (see Figure 1). Likewise, the acid-free leaching system does not have a high redox potential and does not present a favorable copper extraction.

**Figure 16:**
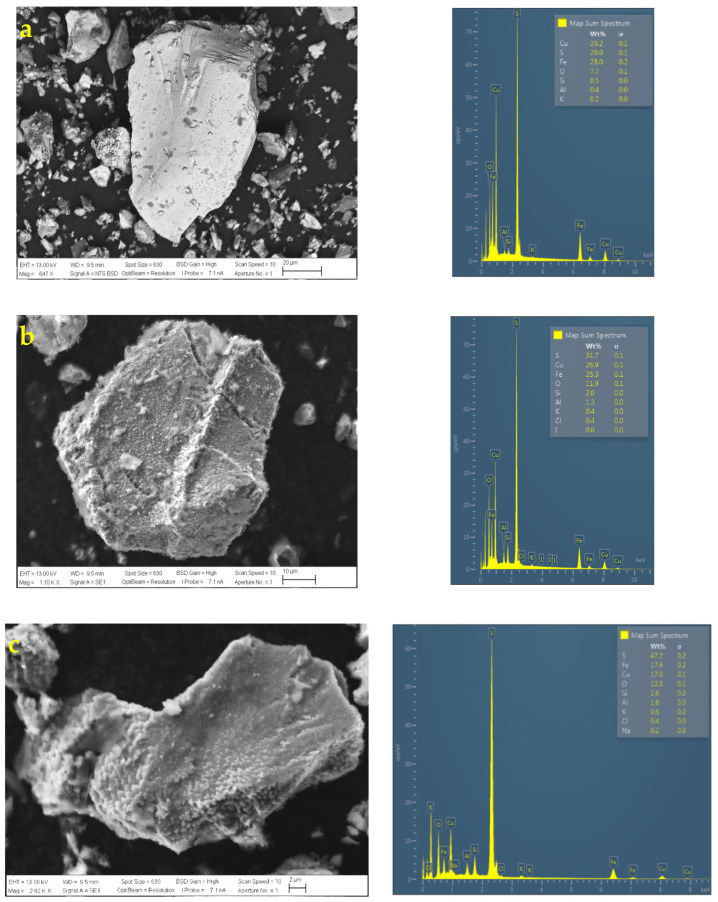
SEM and EDS analysis of chalcopyrite particles with (**a**) H_2_SO_4_ = 0.0 M and KI = 100 ppm, (**b**) H_2_SO_4_ = 0.5 M and KI = 100 ppm, and (**c**) H_2_SO_4_ = 1.0 M and KI = 100 ppm. [Conditions: 45 °C, 50 g of sample with 1000 mL of solution with seawater, 450 rpm].

Figure 16 shows the SEM and EDS analyses performed on the following leach residues:

- H_2_SO_4_ = 1.0 M and KI= 100 ppm;
- H_2_SO_4_ = 0.5 M and KI= 100 ppm;
- H_2_SO_4_ = 0.0 M and KI= 100 ppm.

According to the SEM images, in terms of texture and roughness of the particles, it can be observed that, as the concentration of sulfuric acid varies, the shapes of the particles change. The higher the sulfuric acid concentration, the rougher the surface of the chalcopyrite particles due to the formation of porous sulfur and the presence of precipitates, but, according to the extraction curve, leaching has not yet been inhibited due to the significant number of cracks that the surface of the particles may have, thus continuing the contact between the leaching solution and the mineral.

According to SEM microphotographs, the surface morphology of the chalcopyrite particles is slightly spongy and fractured at acid concentrations of 0.5 and 1.0 M. Without the presence of acid in the leach (see Figure 16a), the surface texture of the chalcopyrite particle is almost intact or without pitting.

In addition, according to EDS analysis, part of the elemental sulfur produced is detected when the copper extraction is higher, which can conclude that part of the sulfide in the bonds that form both pyrite and chalcopyrite is converting to sulfur and precipitates on the surface of the particle but with a spongy morphology. Without the presence of acid (0 M H_2_SO_4_ and 100 ppm KI), it is observed that Cu and Fe remain unleached while, in the case of S, this presents a low value (less than 28 wt. %), which reflects that there is no formation of elemental sulfur on the surface of the particle.

**Figure 17:**
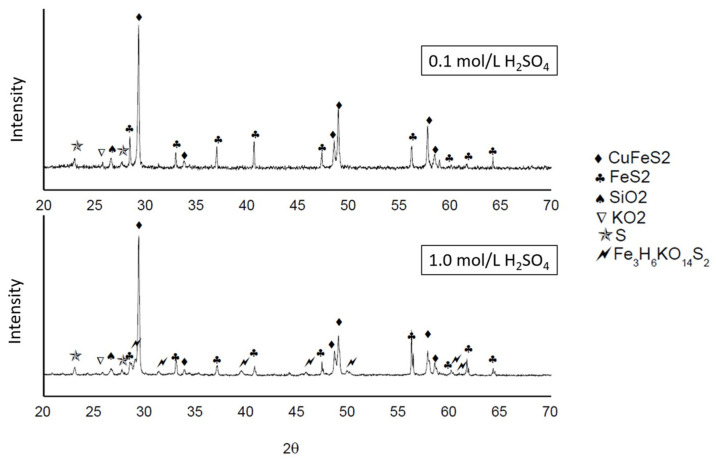
XRD patterns of leaching tests: 0.1 and 1.0 M of H_2_SO_4_ and 100 ppm of KI.

**Figure 18:**
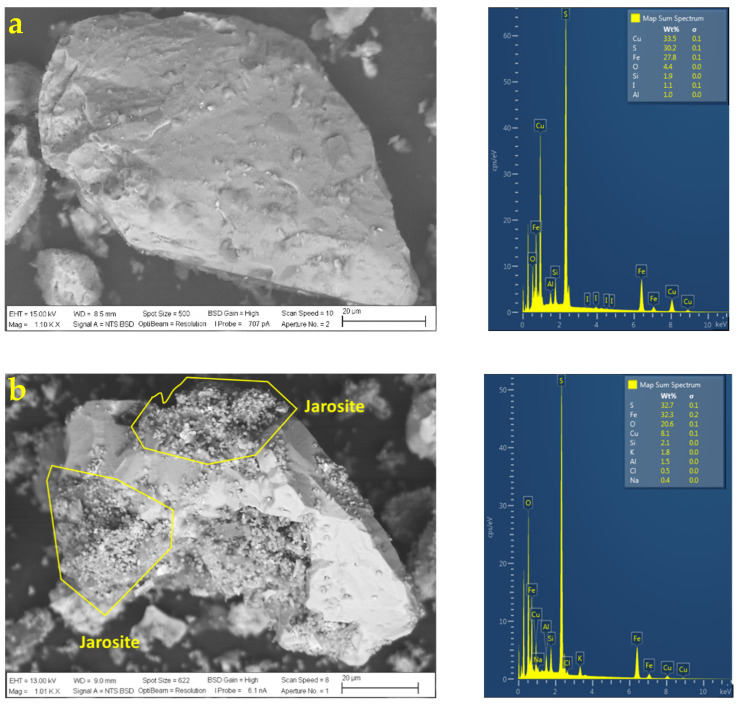
SEM and EDS analyses of chalcopyrite particles with (**a**) H_2_SO_4_ = 0.1 M and (**b**) H_2_SO_4_ = 1.0 M. [Conditions: 100 ppm of KI, 45 °C, 50 g of sample, 1000 mL of solution with seawater, 450 rpm].

Figure 17 shows the respective X-ray diffraction analysis of the samples: (i) 0.1 M of H_2_SO_4_ and 100 ppm of KI and (ii) 1.0 M of H_2_SO_4_ and 100 ppm of KI. The X-ray diffraction patterns show a higher intensity in the peaks belonging to chalcopyrite and pyrite with a minimum concentration of sulfuric acid. On the other hand, a high acid concentration can result in a decrease in the peaks of these minerals. In addition to elemental sulfur, jarosite mineral (Fe_3_H_6_KO_14_S_2_) appears and is also corroborated with the SEM analysis on the chalcopyrite grains (see Figure 18b).

The formation of jarosite precipitates was observed on the surface of chalcopyrite grains leached with 1.0 M H_2_SO_4_. The SEM and EDS analyses (see Figure 18) indicated that the precipitate was mainly composed of Fe, O, S, and K. These elements are one of the precursors of jarosite, which is only stable at low pH values, as it usually occurs in supergene and hypogenic environments by the oxidation of chalcopyrite and pyrite grains or even by oxidation of acidic liquids produced by the reaction with iron-rich rocks [12,60]. Figure 18 also includes a particle leached with 0.1 M H_2_SO_4_ in which no jarosite precipitates are observed (see Figure 18a).

To determine the size and growth of the possible layer formed, either as elemental sulfur or precipitates, the following ripples were analyzed by chord length using the focused beam technique (FBRM):

- H_2_SO_4_ = 0 M and KI= 100 ppm;
- H_2_SO_4_ = 0.1 M and KI= 100 ppm;
- H_2_SO_4_ = 0.5 M and KI= 100 ppm;
- H_2_SO_4_ = 1.0 M and KI= 100 ppm.

**Figure 19:**
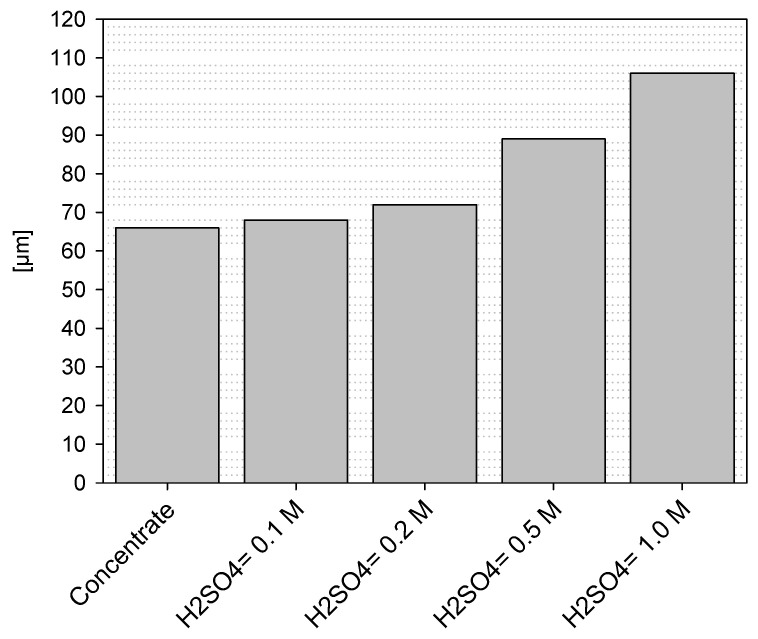
FBRM analysis of the growth of grains belonging to residues using different concentrations of H_2_SO_4_.

According to Figure 19, size changes can be corroborated with the increase in the concentration of sulfuric acid either by precipitation products or the formation of a layer. Higher growth was observed with a concentration of 1.0 mol/L H_2_SO_4_. The growth rate is also corroborated with the SEM analysis on roughness and morphology shown in Figure 16c.

## 4. Conclusions

The leaching of chalcopyrite concentrate was studied using variables such as the concentration of sulfuric acid and iodized salts where, according to the results, iodized salt can be a leaching agent that, at optimal conditions, allows favorable copper extractions. Among the main conclusions obtained according to the results are the following:

- A high concentration of iodide in an acidic medium resulted in low copper extractions, mainly due to the oxidation of a large part of the iodide ions to elemental iodine, which was subsequently released into the environment by sublimation. Low concentrations of iodide ions allowed the presence of iodide ions and triiodide ions in the solution, creating an oxidizing environment. This allows us to conclude that a concentration of 100 ppm of iodide results in better copper extractions.
- In an agitation leaching and an acid medium, the highest copper extraction obtained was approximately 70 % at 100 ppm of KI in 200 h. The high extraction was given by the active state of the chalcopyrite with a redox potential over 600 mV (Ag/AgCl) and a pH less than 2, being in the zones of the ions I_3_^−^ y I^−^.
- A redox potential above 600 mV (Ag/AgCl) has shown that, under these conditions, the rate of chalcopyrite dissolution increases, while at a lower potential, the copper extraction efficiency decreases.
- Concentrations higher than 100 ppm of KIO_3_ improve copper extraction more than using the same concentration of KI.
- The presence of precipitated CuI on chalcopyrite particles occurs when concentrations of 100 ppm of iodide (KI) are used, whereas, at concentrations of 5000 ppm, the presence of CuI is nil, which is assumed to have been released by sublimation.
- Acid is essential to maintain iodide ions with high proton activity. Doses of 1.0 M acid result in the neutralization and production of several layers around the minerals. In addition, high acid concentrations can sulfate the remaining copper. No presence results in extractions of up to about 5% copper where KI is converted into a reducing agent, rather than an oxidizer.
- It was observed that at an acid concentration of 0.5 M, according to the SEM and EDS analyses, part of the sulfur in the bonds forming pyrite and chalcopyrite was transformed into elemental sulfur on the grain surface of the grains, causing the grains to become very rough, with a cracked and spongy morphology. It was also found that at 1.0 M acid, the presence of jarosite became evident.

## Figures and Tables

**Table 1:** Chemical and mineralogical analysis of the chalcopyrite concentrate.

Chemical Analysis		Mineralogical Analysis		
Element	wt. %	Minerals	Formula	wt. %
Si	2.3	Quartz	SiO_2_	2.2
Fe	32.7	Pyrite	FeS_2_	23.3
Al	1.0	Chalcopyrite	CuFeS_2_	61.5
Mg	0.2	Covellite	CuS	1.5
Ca	0.3	Molybdenite	MoS_2_	0.4
Cu	25.0	Dolomite	CaMg(CO_3_)_2_	1.4
Zn	0.5	Boehmite	AlOOH	2.0
Ti	0.5	Chalcanthite	CuSO_4_•5H_2_O	1.2
Mo	0.2	Albite	NaAlSi_3_O_8_	2.2
K	0.4	Muscovite	KAl_2_(Si_3_Al)O_10_(OH)_2_	1.9
C	0.2	Biotite	K(Mg,Fe^2+^)_3_(Si_3_Al)O_10_(OH)_2_	0.7
Na	0.3	Sphalerite	(Zn_x_, Fe_1−x_)S	0.6
S	36.3	Gypsum	CaSO_4_•2H_2_O	0.8
		Clinochlore	(Mg, Fe^2+^)_5_Al(Si_3_Al)O_10_(OH)_8_	0.2

**Table 2:** Major constituents of seawater from San Jorge Bay, Chile (mg L^−1^) [59].

Chemical Method	ICP-AES					AAS		Volumetric Analysis		Gravimetric Analysis
Ionic species	Na^+^	Mg^2+^	Ca^2+^	K^+^	B^3+^	Cu^2+^	NO_3_^−^	Cl^−^	HCO_3_^−^	SO_4_^2−^
mg·L^−1^	9480	1190	386	374	4.6	0.07	2.05	18,765	142	2771

## Data Availability

The data presented in this study are available on request from authors C.I.C and M.E.T.

## References

[1] Wang H.-D., Li Y.-B., Wang B., Wang X.-Y. (2018). Fundamental Study of Different Impurity Ions on Chalcopyrite Leaching Process. Met. Mine.

[2] Lim Y.H., Kim S.H., Lee H.I., Jung K.B., Yoo K. (2019). Leaching of copper from chalcopyrite using 50 L pressure oxidation autoclave. J. Korean Soc. Miner. Energy Resour. Eng..

[3] Mokmeli M. (2020). Pre feasibility study in hydrometallurgical treatment of low-grade chalcopyrite ores from Sarcheshmeh copper mine. Hydrometallurgy.

[4] Mokmeli M., Parizi M.T. (2022). Low-grade chalcopyrite ore, heap leaching or smelting recovery route?. Hydrometallurgy.

[5] Watling H. (2006). The bioleaching of sulphide minerals with emphasis on copper sulphides—A review. Hydrometallurgy.

[6] Sokić M., Marković B., Stanković S., Kamberović Ž., Štrbac N., Manojlović V., Petronijević N. (2019). Kinetics of chalcopyrite leaching by hydrogen peroxide in sulfuric acid. Metals.

[7] Shin D., Ahn J., Lee J. (2019). Kinetic study of copper leaching from chalcopyrite concentrate in alkaline glycine solution. Hydrometallurgy.

[8] Watling H. (2013). Chalcopyrite hydrometallurgy at atmospheric pressure: 1. Review of acidic sulfate, sulfate–chloride and sulfate–nitrate process options. Hydrometallurgy.

[9] Wang S. (2005). Copper leaching from chalcopyrite concentrates. JOM.

[10] Habashi F. (1978). Chalcopyrite: Its Chemistry and Metallurgy.

[11] Zhao H., Zhang Y., Zhang X., Qian L., Sun M., Yang Y., Zhang Y., Wang J., Kim H., Qiu G. (2019). The dissolution and passivation mechanism of chalcopyrite in bioleaching: An overview. Miner. Eng..

[12] Kartal M., Xia F., Ralph D., Rickard W.D., Renard F., Li W. (2020). Enhancing chalcopyrite leaching by tetrachloroethylene-assisted removal of sulphur passivation and the mechanism of jarosite formation. Hydrometallurgy.

[13] Yang B., Luo W., Wang X., Yu S., Gan M., Wang J., Liu X., Qiu G. (2020). The use of biochar for controlling acid mine drainage through the inhibition of chalcopyrite biodissolution. Sci. Total Environ..

[14] Panda S., Sanjay K., Sukla L., Pradhan N., Subbaiah T., Mishra B., Prasad M., Ray S. (2012). Insights into heap bioleaching of low grade chalcopyrite ores—A pilot scale study. Hydrometallurgy.

[15] Kelly R.G., Scully J.R., Shoesmith D., Buchheit R.G. (2002). Electrochemical Techniques in Corrosion Science and Engineering.

[16] Uhlig H.H. (1979). Passivity in metals and alloys. Corros. Sci..

[17] O’Connor G., Eksteen J. (2020). A critical review of the passivation and semiconductor mechanisms of chalcopyrite leaching. Miner. Eng..

[18] Dutrizac J. (1990). Elemental sulphur formation during the ferric chloride leaching of chalcopyrite. Hydrometallurgy.

[19] Lv C., Wu H., Lin W., Illerup J.B., Karcz A.P., Ye S., Damø A.J. (2019). Characterization of elemental sulfur in chalcopyrite leach residues using simultaneous thermal analysis. Hydrometallurgy.

[20] Gomes B.L.F.d.M., Bertoli A.C., Duarte H.A. (2022). Growing Mechanism of Polysulfides and Elemental Sulfur Formation: Implications to Hindered Chalcopyrite Dissolution. J. Phys. Chem. A.

[21] Nourmohamadi H., Esrafili M.D., Aghazadeh V. (2021). DFT study of ferric ion interaction with passive layer on chalcopyrite surface: Elemental sulfur, defective sulfur and replacement of M2+ (M= Cu and Fe) ions. Comput. Condens. Matter.

[22] Velásquez-Yévenes L., Nicol M., Miki H. (2010). The dissolution of chalcopyrite in chloride solutions Part 1. The effect of solution potential. Hydrometallurgy.

[23] de Oliveira C., Duarte H.A. (2010). Disulphide and metal sulphide formation on the reconstructed (0 0 1) surface of chalcopyrite: A DFT study. Appl. Surf. Sci..

[24] Parker G.K., Woods R., Hope G.A. (2008). Raman investigation of chalcopyrite oxidation. Colloids Surf. A Physicochem. Eng. Asp..

[25] Liang C., Xia J., Yang Y., Nie Z., Qiu G. (2012). Progress in sulfur speciation transformation during chalcopyrite bioleaching. Chin. J. Nonferrous Met..

[26] Majuste b., Ciminelli V., Osseo-Asare K., Dantas M., Magalhães-Paniago R. (2012). Electrochemical dissolution of chalcopyrite: Detection of bornite by synchrotron small angle X-ray diffraction and its correlation with the hindered dissolution process. Hydrometallurgy.

[27] Klauber C. (2008). A critical review of the surface chemistry of acidic ferric sulphate dissolution of chalcopyrite with regards to hindered dissolution. Int. J. Miner. Process..

[28] Parker A., Klauber C., Kougianos A., Watling H., Van Bronswijk W. (2003). An X-ray photoelectron spectroscopy study of the mechanism of oxidative dissolution of chalcopyrite. Hydrometallurgy.

[29] Córdoba E., Muñoz J., Blázquez M., González F., Ballester A. (2009). Passivation of chalcopyrite during its chemical leaching with ferric ion at 68 C. Miner. Eng..

[30] Carrillo-Pedroza F., Sánchez-Castillo M., Soria-Aguilar M., Martínez-Luévanos A., Gutiérrez E. (2010). Evaluation of acid leaching of low grade chalcopyrite using ozone by statistical analysis. Can. Metall. Q..

[31] Wang J., Faraji F., Ghahreman A. (2021). Evaluation of ozone as an efficient and sustainable reagent for chalcopyrite leaching: Process optimization and oxidative mechanism. J. Ind. Eng. Chem..

[32] Havlik T., Skrobian M. (1990). Acid leaching of chalcopyrite in the presence of ozone. Can. Metall. Q..

[33] Petrović S.J., Bógdanović G.D., Antonijević M.M. (2018). Leaching of chalcopyrite with hydrogen peroxide in hydrochloric acid solution. Trans. Nonferrous Met. Soc. China.

[34] Turan M.D., Sarı Z.A., Nizamoğlu H. (2021). Pressure leaching of chalcopyrite with oxalic acid and hydrogen peroxide. J. Taiwan Inst. Chem. Eng..

[35] Moraga G.A., Jamett N.E., Hernández P.C., Graber T.A., Taboada M.E. (2021). Chalcopyrite Leaching with Hydrogen Peroxide and Iodine Species in Acidic Chloride Media at Room Temperature: Technical and Economic Evaluation. Metals.

[36] Antonijević M., Janković Z., Dimitrijević M. (1994). Investigation of the kinetics of chalcopyrite oxidation by potassium dichromate. Hydrometallurgy.

[37] Aydogan S., Ucar G., Canbazoglu M. (2006). Dissolution kinetics of chalcopyrite in acidic potassium dichromate solution. Hydrometallurgy.

[38] Shiers D., Collinson D., Kelly N., Watling H. (2016). Copper extraction from chalcopyrite: Comparison of three non-sulfate oxidants, hypochlorous acid, sodium chlorate and potassium nitrate, with ferric sulfate. Miner. Eng..

[39] Hackl R., Dreisinger D., Peters E., King J. (1995). Passivation of chalcopyrite during oxidative leaching in sulfate media. Hydrometallurgy.

[40] Cháidez J., Parga J., Valenzuela J., Carrillo R., Almaguer I. (2019). Leaching chalcopyrite concentrate with oxygen and sulfuric acid using a low-pressure reactor. Metals.

[41] Mojtahedi B., Rasouli S., Yoozbashizadeh H. (2020). Pressure leaching of chalcopyrite concentrate with oxygen and kinetic study on the process in sulfuric acid solution. Trans. Indian Inst. Met..

[42] Anjum F., Shahid M., Akcil A. (2012). Biohydrometallurgy techniques of low grade ores: A review on black shale. Hydrometallurgy.

[43] Vakylabad A.B., Schaffie M., Naseri A., Ranjbar M., Manafi Z. (2016). Optimization of staged bioleaching of low-grade chalcopyrite ore in the presence and absence of chloride in the irrigating lixiviant: ANFIS simulation. Bioprocess Biosyst. Eng..

[44] Schlesinger M.E., Sole K.C., Davenport W.G., Alvear G.R. (2021). Extractive Metallurgy of Copper.

[45] Forward F., Warren I. (1960). Extraction of metals from sulphide ores by wet methods. Metall. Rev..

[46] Yannopoulos J.C. (2012). The Extractive Metallurgy of Gold.

[47] Syed S. (2012). Recovery of gold from secondary sources—A review. Hydrometallurgy.

[48] Davis A., Tran T., Young D. (1993). Solution chemistry of iodide leaching of gold. Hydrometallurgy.

[49] Konyratbekova S., Baikonurova A., Ussoltseva G., Erust C., AKCIL A. (2015). Thermodynamic and kinetic of iodine–iodide leaching in gold hydrometallurgy. Trans. Nonferrous Met. Soc. China.

[50] Wang L., Yin S., Deng B., Wu A. (2022). Copper sulfides leaching assisted by acidic seawater-based media: Ionic strength and mechanism. Miner. Eng..

[51] Winarko R., Dreisinger D.B., Miura A., Tokoro C., Liu W. (2020). Kinetic modelling of chalcopyrite leaching assisted by iodine in ferric sulfate media. Hydrometallurgy.

[52] Granata G., Miura A., Liu W., Pagnanelli F., Tokoro C. (2019). Iodide-assisted leaching of chalcopyrite in acidic ferric sulfate media. Hydrometallurgy.

[53] Fukano Y., Miura A. (2021). Chalcopyrite leaching with iodine (JX Iodine Process) for various ore types. Hydrometallurgy.

[54] Winarko R., Dreisinger D.B., Miura A., Fukano Y., Liu W. (2022). Iodine-assisted chalcopyrite leaching in ferric sulfate media: Kinetic study under fully controlled redox potential and pH. Hydrometallurgy.

[55] Allen T., Keefer R. (1955). The formation of hypoiodous acid and hydrated iodine cation by the hydrolysis of iodine. J. Am. Chem. Soc..

[56] Manabe M. (2012). Method of Leaching Copper Sulfide Ore with the Use of Iodine. U.S. Patent.

[57] Moreno L., Ordóñez J., Cisternas L. (2014). La industria salitrera y los recursos hídricos. Seawater in Mining.

[58] Ordóñez J.I., Moreno L., Mellado M.E., Cisternas L.A. (2014). Modeling validation of caliche ore leaching using seawater. Int. J. Miner. Process..

[59] Torres C., Taboada M., Graber T., Herreros O., Ghorbani Y., Watling H. (2015). The effect of seawater based media on copper dissolution from low-grade copper ore. Miner. Eng..

[60] Stoffregen R., Alpers C., Jambor J. (2001). Alunite-Jarosite Crystallography, Thermodynamics, and Geochronology. Rev. Mineral. Geochem..

[61] Koleini S.J., Aghazadeh V., Sandström Å. (2011). Acidic sulphate leaching of chalcopyrite concentrates in presence of pyrite. Miner. Eng..

[62] You L.Q., Heping L., Li Z. (2007). Study of galvanic interactions between pyrite and chalcopyrite in a flowing system: Implications for the environment. Environ. Geol..

[63] Cruz R., Luna-Sánchez R., Lapidus G., González I., Monroy M. (2005). An experimental strategy to determine galvanic interactions affecting the reactivity of sulfide mineral concentrates. Hydrometallurgy.

[64] Holmes P., Crundwell F. (1995). Kinetic aspects of galvanic interactions between minerals during dissolution. Hydrometallurgy.

[65] Peng Y., Grano S., Fornasiero D., Ralston J. (2003). Control of grinding conditions in the flotation of chalcopyrite and its separation from pyrite. Int. J. Miner. Process..

[66] Yepsen Ferreira O.G. (2018). Evaluación de procesos de oxidación avanzados en el procesamiento de minerales sulfurados y concentrados de cobre. Ph.D. Thesis.

[67] Free M. Understanding acid consumption and its relationship with copper recovery. Proceedings of the SME Annual Meeting.

[68] Snäll S., Liljefors T. (2000). Leachability of major elements from minerals in strong acids. J. Geochem. Explor..

[69] Chetty D. (2018). Acid-gangue interactions in heap leach operations: A review of the role of mineralogy for predicting ore behaviour. Minerals.

[70] Hernández P., Dorador A., Martínez M., Toro N., Castillo J., Ghorbani Y. (2020). Use of seawater/brine and caliche’s salts as clean and environmentally friendly sources of chloride and nitrate ions for chalcopyrite concentrate leaching. Minerals.

[71] Hernández P., Taboada M., Herreros O., Graber T., Ghorbani Y. (2018). Leaching of Chalcopyrite in Acidified Nitrate Using Seawater-Based Media. Minerals.

[72] Castellón C.I., Hernández P.C., Velásquez-Yévenes L., Taboada M.E. (2020). An Alternative Process for Leaching Chalcopyrite Concentrate in Nitrate-Acid-Seawater Media with Oxidant Recovery. Metals.

[73] Yévenes L.V., Miki H., Nicol M. (2010). The dissolution of chalcopyrite in chloride solutions: Part 2: Effect of various parameters on the rate. Hydrometallurgy.

[74] Nicol M., Miki H., Velásquez-Yévenes L. (2010). The dissolution of chalcopyrite in chloride solutions: Part 3. Mechanisms. Hydrometallurgy.

[75] Córdoba E., Muñoz J., Blázquez M., González F., Ballester A. (2008). Leaching of chalcopyrite with ferric ion. Part II: Effect of redox potential. Hydrometallurgy.

[76] Kametani H., Aoki A. (1985). Effect of suspension potential on the oxidation rate of copper concentrate in a sulfuric acid solution. Metall. Trans. B.

[77] Rivera-Velasquez B., Viramontes-Gamboa G., Dixon D. Leaching of chalcopyrite concentrates in acid ferric sulfate media at controlled redox potential. Proceedings of the HydroProcess 2006, International Workshop on Process Hydrometallurgy.

[78] Sandström Å., Shchukarev A., Paul J. (2005). XPS characterisation of chalcopyrite chemically and bio-leached at high and low redox potential. Miner. Eng..

[79] Viramontes-Gamboa G., Peña-Gomar M.M., Dixon D.G. (2010). Electrochemical hysteresis and bistability in chalcopyrite passivation. Hydrometallurgy.

[80] Viramontes-Gamboa G., Rivera-Vasquez B.F., Dixon D.G. (2007). The active-passive behavior of chalcopyrite: Comparative study between electrochemical and leaching responses. J. Electrochem. Soc..

[81] Muraleedharan K., Kannan M., Ganga D.T. (2011). Thermal decomposition kinetics of potassium iodate. J. Therm. Anal. Calorim..

[82] Xiong X., Hua X., Zheng Y., Lu X., Li S., Cheng H., Xu Q. (2018). Oxidation mechanism of chalcopyrite revealed by X-ray photoelectron spectroscopy and first principles studies. Appl. Surf. Sci..

[83] Cox E.M., Arai Y. (2014). Environmental chemistry and toxicology of iodine. Advances in Agronomy.

[84] Brent Hiskey J., Atluri V. (1988). Dissolution chemistry of gold and silver in different lixiviants. Miner. Procesing Extr. Metall. Rev..

[85] Konyratbekova S.S., Baikonurova A., Akcil A. (2015). Non-cyanide leaching processes in gold hydrometallurgy and iodine-iodide applications: A review. Miner. Process. Extr. Metall. Rev..

[86] Rancon D. (1988). Comparative study of radioactive iodine behavior in soils under various experimental and natural conditions. Radiochim. Acta.

[87] Nikoloski A.N., O’Malley G.P. (2018). The acidic ferric sulfate leaching of primary copper sulfides under recycle solution conditions observed in heap leaching. Part 1. Effect of standard conditions. Hydrometallurgy.

[88] Nicol M., Miki H., Basson P. (2013). The effects of sulphate ions and temperature on the leaching of pyrite. 2. Dissolution rates. Hydrometallurgy.

